# Whole-Genome Sequencing Reveals Genetic Variation in the Asian House Rat

**DOI:** 10.1534/g3.116.029504

**Published:** 2016-04-27

**Authors:** Huajing Teng, Yaohua Zhang, Chengmin Shi, Fengbiao Mao, Lingling Hou, Hongling Guo, Zhongsheng Sun, Jianxu Zhang

**Affiliations:** * The State Key Laboratory of Integrated Management of Pest Insects and Rodents, Institute of Zoology, Chinese Academy of Sciences, 100101 Beijing, China; † University of Chinese Academy of Sciences, 100049 Beijing, China; ‡ Beijing Institutes of Life Science, Chinese Academy of Sciences, 100101, China,; § Beijing Institute of Genomics, Chinese Academy of Sciences, 100101 Beijing, China

**Keywords:** genetic landscape, next-generation sequencing, *Rattus tanezumi*, single-nucleotide polymorphisms, structural variations

## Abstract

Whole-genome sequencing of wild-derived rat species can provide novel genomic resources, which may help decipher the genetics underlying complex phenotypes. As a notorious pest, reservoir of human pathogens, and colonizer, the Asian house rat, *Rattus tanezumi*, is successfully adapted to its habitat. However, little is known regarding genetic variation in this species. In this study, we identified over 41,000,000 single-nucleotide polymorphisms, plus insertions and deletions, through whole-genome sequencing and bioinformatics analyses. Moreover, we identified over 12,000 structural variants, including 143 chromosomal inversions. Further functional analyses revealed several fixed nonsense mutations associated with infection and immunity-related adaptations, and a number of fixed missense mutations that may be related to anticoagulant resistance. A genome-wide scan for loci under selection identified various genes related to neural activity. Our whole-genome sequencing data provide a genomic resource for future genetic studies of the Asian house rat species and have the potential to facilitate understanding of the molecular adaptations of rats to their ecological niches.

Genetic variations of the laboratory rat have served as a valuable resource in biomedical and behavioral research for nearly 200 yr (Baker *et al.* 1979; Jacob 1999). However, the extant strains of laboratory rat originate from limited *Rattus norvegicus* founder populations (Baker *et al.* 1979; Krinke 2000; Smits *et al.* 2005; Saar *et al.* 2008). Extensive genetic variation is required to facilitate correlation of genotypes with complex behavioral or ecologically relevant traits. Wild-derived rat strains or species can provide novel genome resources to decipher the genetic mechanisms underlying such complex phenotypes (Smits *et al.* 2005).

As a diploid species of rodent in the genus *Rattus*, the Asian house rat (AHR, *R. tanezumi*; 2n = 42) (Hamta *et al.* 2006; Badenhorst *et al.* 2011) is widely distributed in Asia, and has recently invaded the South Pacific, African countries, and the United States (Wilson and Reeder 2005; Bastos *et al.* 2011; Lack *et al.* 2012; Conroy *et al.* 2013; Nakayama *et al.* 2013). As an agricultural pest (Singleton *et al.* 2007; Huang *et al.* 2013), the AHR demonstrates superior adaptive potential, compared to other rodents, with higher resistance to commonly used rodenticides (Zheng 1981; Tian *et al.* 2006; Huang *et al.* 2012). The AHR can also serve as a reservoir of a wide range of pathogens associated with human disease (Plyusnina *et al.* 2009; Jonsson *et al.* 2010; Yin *et al.* 2011; Kocher *et al.* 2015). However, although the AHR represents a tremendous threat to agriculture and human health, little is known about the genetic basis underlying its remarkable biological traits. A comprehensive genetic inventory of the AHR will not only be essential for the development of effective pest control programs, but will also advance our understanding of the diversity of rodent-borne zoonosis.

Rapid advances in next-generation sequencing (NGS) technology and reverse ecology hold great promise for deciphering the genetic basis underlying the functional variation of natural organisms (Ellison *et al.* 2011; Liu *et al.* 2014). In the present study, we used an NGS-based pooled sequencing strategy to analyze genome-wide variation, including single-nucleotide polymorphisms (SNPs) and structural variations (SVs), across the AHR genome. Our data provide a resource for future population genetic research on the AHR, and advance our understanding of the molecular adaptations of rats with respect to their ecological niches.

## Materials and Methods

### Phylogenetic analysis of the AHR based on mitochondrial genomes

**Figure 1:**
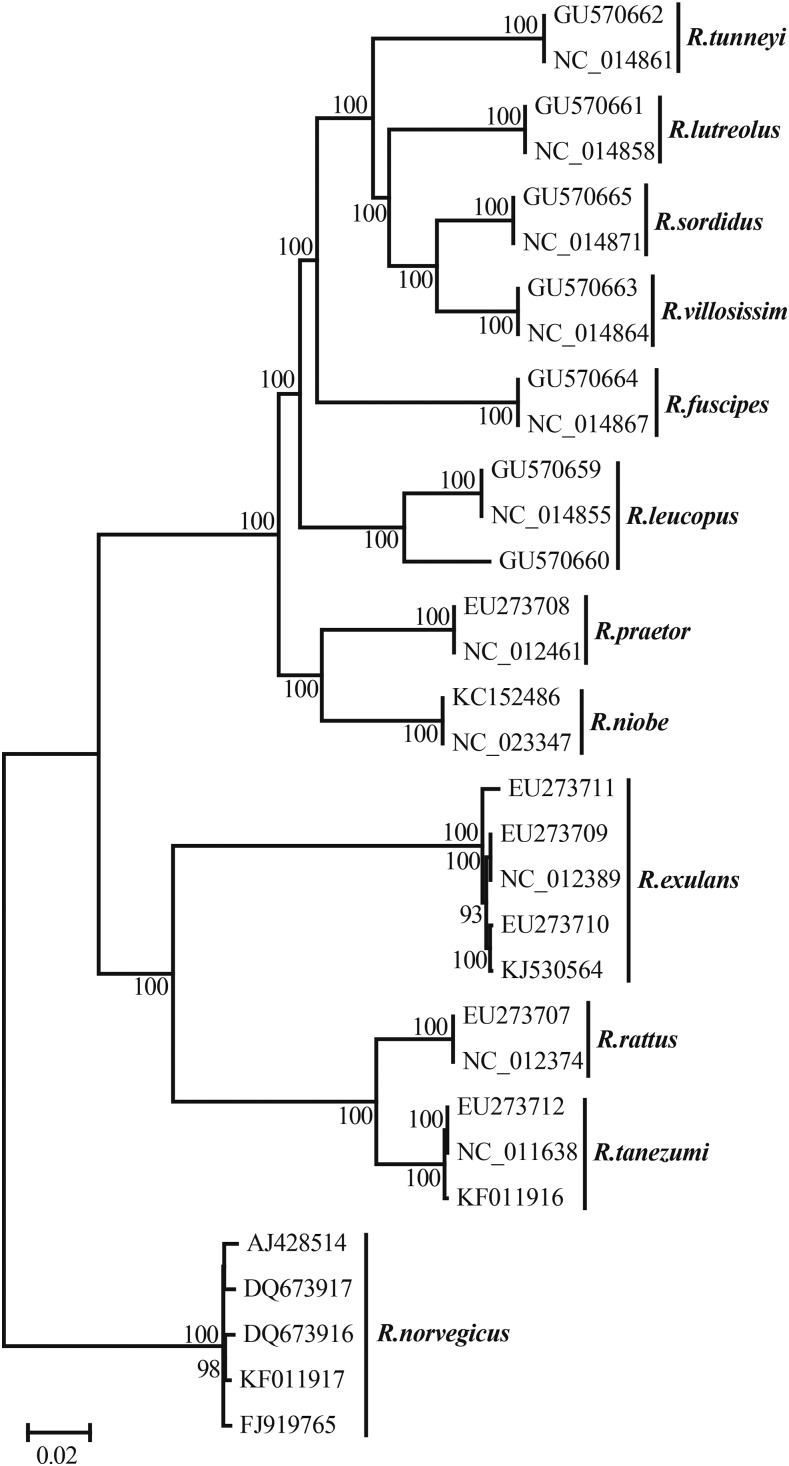
Phylogenetic tree of *Rattus* spp., estimated using mitochondrial genomes. Published mitochondrial genome sequences of the *Rattus* genus were downloaded from GenBank and Accession numbers are indicated.

To estimate the phylogenetic position of the AHR, published *Rattus* genus mitochondrial genome sequences (accession numbers listed in Figure 1) were downloaded from GenBank. The sequences of 13 mitochondrial protein-coding genes were joined and aligned using MUSCLE (Edgar 2004). The best mutational model (GTR+I+G) was then selected using jModelTest v2.1.7 (Darriba *et al.* 2012). Mitochondrial genome phylogenetic analyses were conducted using MrBayes v3.22 (Ronquist *et al.* 2012), with two independent analyses running in parallel, each with four Markov Chain Monte Carlo (MCMC) chains. The final tree topology was recovered after a total of 10,000,000 generations, sampling 1 in every 10,000 generations after discarding the first 1000 as burn-in.

### Samples, DNA extraction, and library preparation

In 2014, 42 individual AHRs, including 24 from Dujiangyan (31°01’N/103°40’E), Sichuan Province, China and 18 from Taiyuan (37°43’N/112°29’E), Shanxi Province, China, were trapped and identified using mitochondrial cytochrome oxidase subunit I barcode sequences. Genomic DNA samples were extracted from small pieces of tail using a TailGen DNA extraction Kit (CWBIO, Beijing, China). The quality and integrity of the extracted DNA was checked by measuring the A260/A280 ratio using a NanoDrop ND-1000 spectrophotometer (Thermo Fisher Scientific Inc., Waltham, MA) and by agarose gel electrophoresis. Then, equivalent amounts of DNA extracted from all individuals were pooled for library construction. A library with an insert size of approximately 300 bp was prepared and sequenced on either an Illumina HiSequation 2000 or an X Ten instrument, with 100 or 150 bp paired-end reads, respectively. After filtering out of raw sequencing reads containing adapters and reads of low quality, the remaining clean reads were aligned to the reference genome of *R. norvegicus* (RGSC5.0, Ensembl release 70), a close relative of AHR, using the Burrows-Wheeler Alignment tool (BWA v0.7.5a-r405) with default parameters (Li and Durbin 2009). Genome size was estimated on the basis of k-mer frequency distribution using KmerGenie with default parameters (Chikhi and Medvedev 2014). Duplicate reads were removed using the rmdup function in SAMtools v 0.1.19-44428cd (Li *et al.* 2009). In addition, unmapped reads were assembled *de novo* using SOAPdenovo v2.04 (Luo *et al.* 2012) and gene predictions were performed using AUGUSTUS (Hoff and Stanke 2013).

### Small variant calling and genetic diversity estimation

After performing the alignment and removing duplicate reads, two different variant calling tools, SNVer v0.5.3 (Wei *et al.* 2011) and VarScan v2.3.7 (Koboldt *et al.* 2009; Koboldt *et al.* 2012), were used to detect SNPs and small insertions and deletions (InDels) in the pooled samples. For SNVer, we set the number of haploids to 84, and used the RGSC v5.0 reference assembly with the Single-Nucleotide Polymorphism database (dbSNP) build 136. Prior to running VarScan, a pileup file was created using the mpileup command in SAMtools. Then, variants were called using the VarScan subcommands mpileup2snp and mpileup2indel with the following parameters: –min-coverage 20, –min-avg-qual 20, and –p-value 1e-02. In our further analyses, we considered only those SNPs and small InDels that were obtained using both pipelines.

Two summary statistics, nucleotide diversity (π) (Nei 1987) and Watterson’s theta (θ_W_) (Watterson 1975), are commonly used for measuring genetic diversity within a population (Tajima 1983). We used the PoPoolation package (Kofler *et al.* 2011; Schlotterer *et al.* 2014) to estimate π and θ_W_ in sliding windows across the genome, with a window size of 100 kb and a step size of 20 kb, and the following parameters: –min-coverage 20, –max-coverage 500, –min-qual 20, and –pool-size 42.

### Structural variation detection

Following the removal of duplicate reads, large InDels and inversions were detected using MetaSV v0.5 (Mohiyuddin *et al.* 2015) on the alignment to the RGSC v5.0 reference genome. The integrative structural-variant caller MetaSV merges results obtained by multiple detection methods, including BreakDancer (Chen *et al.* 2009), LUMPY (Layer *et al.* 2014), and Pindel (Ye *et al.* 2009). BreakDancer can detect a cluster of reads with abnormal length of insert size, or incorrect orientation of ends, based on paired-end read mapping. In contrast, Pindel splits the unmapped end of a one-end anchored read into a few pieces, and performs local realignment of each piece in the candidate region. Finally, LUMPY incorporates both split read analysis and read-pair discordance to detect breakpoints. To evaluate the accuracy of the predicted breakpoints, 20 randomly selected deletion and inversion breakpoints were analyzed using polymerase chain reaction (PCR)-based Sanger sequencing with the primers listed in Supplemental Material, File S1. Inversions were displayed using RCircos (Zhang *et al.* 2013) and inGAP-sv (Qi and Zhao 2011).

### Variant annotation and identification of neutral SNPs

SNPEff v4.0e (Cingolani *et al.* 2012) was used to annotate the identified SNPs based on the Rnor5.0.74 rat assembly. Our previously sequenced individual genome of the AHR sibling species, *R. rattus*, was also reanalyzed and annotated (Deinum *et al.* 2015). Fourfold degenerate sites have traditionally been regarded as neutral variations in mammals, due to the degenerate nature of the genetic code (Eyre-Walker and Keightley 1999; Nachman and Crowell 2000). Because of the uncertainty of the nature of predicted SNPs in segmental duplications (Fredman *et al.* 2004), SNPs residing in duplicated genes were removed from the heterozygous fourfold degenerate dataset in order to obtain reliable neutral SNPs. Furthermore, in order to exclude linkage between SNPs, the distance between neighboring neutral SNPs was set to at least 100,000 bp.

### Identification of selection footprints and functional characterization of selected genes

To identify footprints of selection in the AHR genome, we estimated genome-wide allele frequencies using the Pool_hmm program (Boitard *et al.* 2009, 2012, 2013) with the following options: -c 20, –q 20, -k 1e-10, –pred, and –theta 0.0018. Pool_hmm estimates allele frequencies at each polymorphic site based on a probabilistic model, and different patterns of allele frequencies are associated with different hidden states: “Neutral”, “Intermediate”, and “Selection”. The top 5% of polymorphic sites, based on the posterior probability of the hidden state “Selection”, were selected, and neighboring loci were merged and identified as positively selected regions. Candidate sweep regions were further identified based on the fact that focal regions show reduced nucleotide diversity in the AHR population (the bottom 5% quantile of the mean genome-wide distribution). To characterize the molecular functions of the genes contained in selective sweep regions, we performed functional enrichment analyses using the clusterProfiler toolkit (Yu *et al.* 2012).

### Data availability

The raw sequencing datasets were deposited in the Sequence Read Archive (http://www.ncbi.nlm.nih.gov/sra/) under accession numbers SRX1425877 and SRX1425879. Table S1 and Table S2 contain nonsense mutations and gene ontology information, respectively, for genes with frameshifts in the AHR. BLASTP results detailing predicted proteins for the *de novo* assembled unmapped reads are listed in Table S3. Genes in selective sweep regions, and functional annotation of selective sweep regions of the AHR genome, are listed in Table S4 and Table S5, respectively. File S1 contains PCR-based Sanger sequencing of the candidate structural variation breakpoints. Fixed missense mutations in the warfarin interaction pathway and polymorphic and unlinked neutral sites of single-copy protein-coding genes are listed in File S2 and File S3, respectively. Frameshift variants and SVs of the AHR are listed in File S4 and File S5, respectively.

## Results

### Whole-genome sequencing and mapping

**Figure 2:**
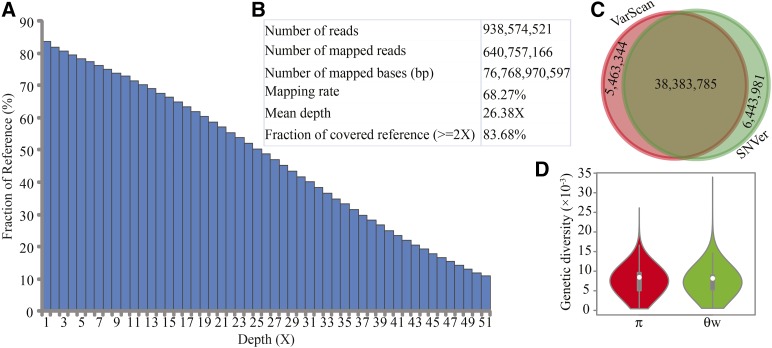
Genetic diversity in the AHR. Mapping rate, depth, and genome coverage of AHR sequencing data (A, B); overlap of SNPs obtained from two different callers, VarScan and SNVer (C); and distribution of summary statistics: nucleotide diversity (π) and Watterson’s theta (θ_W_) (D).

More than 938 million clean reads, corresponding to 112 Gb of sequencing data, were generated. The genome size of the AHR was approximately 3.28 Gb estimated based on the k-mer frequency distribution. Approximately 68.27% of the sequencing data could be mapped to the reference genome of the closely related species, *R. norvegicus*, although the two species diverged more than 2.2 million yr ago (Figure 1) (Robins *et al.* 2008, 2010). Approximately 83.68% of the reference genome was covered by at least two reads, the effective genome-wide average sequencing depth was 26.38-fold, and the percentage of the genome with a depth of 10 or more was 73.99% (Figure 2). In an attempt to identify novel genomic sequences, we assembled unmapped reads and obtained 2979 scaffolds with a minimum length of 1 kb and a total of 3.42 Mb in length.

### SNP calling

**Figure 3:**
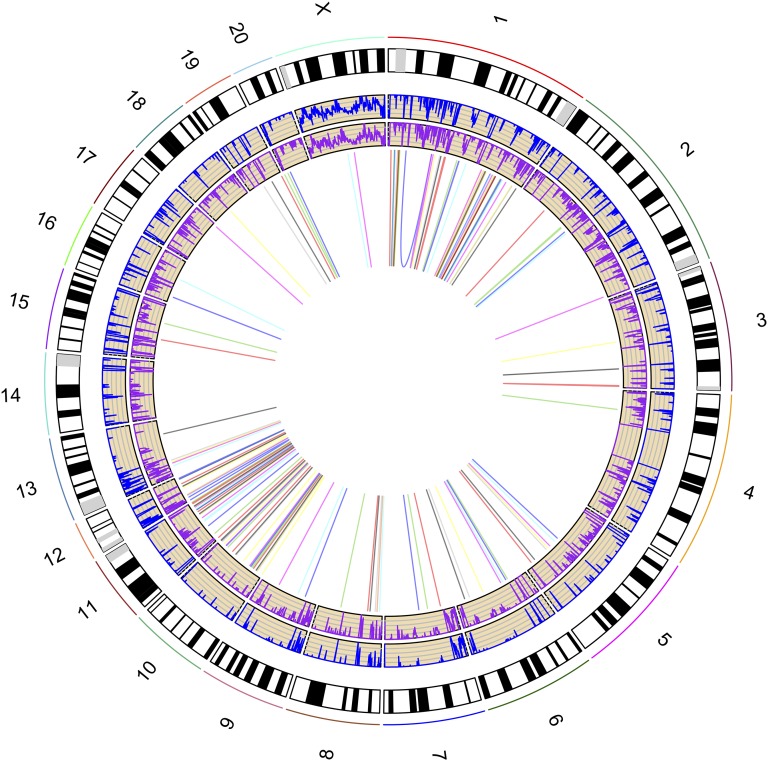
Distribution of SNPs, InDels, and inversions in the AHR genome relative to *R. norvegicus* chromosomes and karyotypes. The innermost circle shows inversions. The next circles show lines representing small InDels (purple) and SNP density (blue).

**Figure 4:**
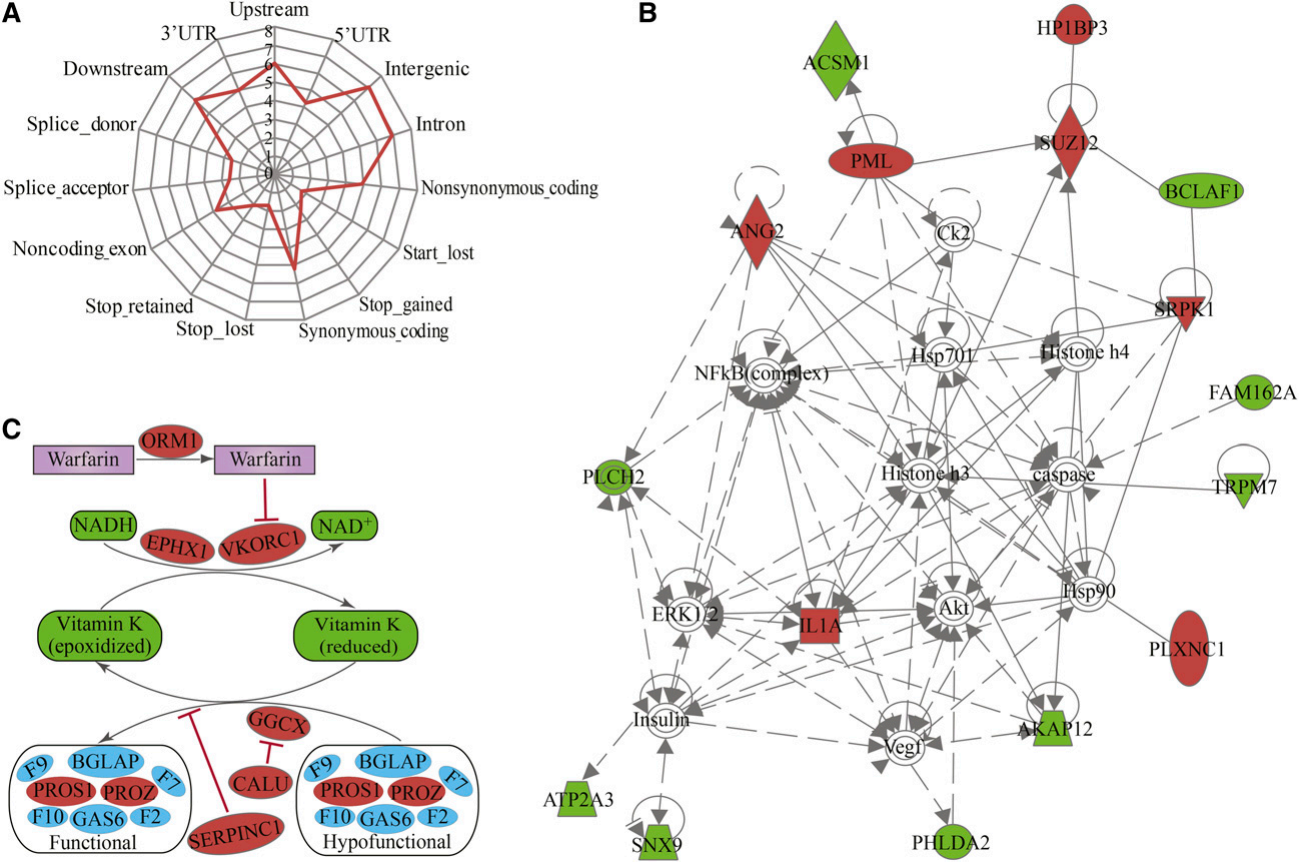
Functional significance of genetic variants in the AHR. (A) Annotation of homozygous AHR SNPs. (B) Interaction network analysis of proteins containing nonsense mutations using the Ingenuity Pathway Knowledge Base (IPKB). (C) Missense-mutated genes of the warfarin interaction pathway. (A) The axes in the radar chart represent annotated genomic features and Log_10_-transformed values. (B) Color shading corresponds to identified genes containing nonsense mutations (Table S1), with red indicating viral-infection-related genes. Direct (solid lines) and indirect (dashed lines) interactions of genes are based on the IPKB database. The shape of nodes indicates the major function of the protein. (C) Depicts simplified interrelationships between vitamin K, coagulation factors (II, VII, IX, and X), and warfarin. The identified missense-mutated genes of the warfarin interactive pathway (File S2) are indicated in red.

A total of 38,383,785 SNPs were identified in the AHR genome compared with the reference genome (Figure 2 and Figure 3). The means of the summary statistics, π and θ_W_, were 7.133 × 10^−3^ and 6.887 × 10^−3^, respectively (Figure 2), suggesting more sequence diversity in the AHR than was reported for *R. norvegicus* (Ness *et al.* 2012). Among the SNPs, 49.85% (19,134,953) were homozygous, and were thus regarded as fixed variants in the AHR in our study. Among the fixed SNPs, 250,606 were located in protein-coding regions, and 82,495 and 167,681 of these were nonsynonymous and synonymous variants, respectively (Figure 4). Of the 82,495 nonsynonymous variants, 239 were predicted to cause premature truncation of proteins due to the insertion of stop codons, whereas 51 were predicted to cause the removal of stop codons (Table S1 and Figure 4). Of the 239 nonsense mutations, 106 were shared by the AHR sibling species, *R. rattus*, another pathogen carrier (Meerburg *et al.* 2009; Himsworth *et al.* 2015), suggesting a possible ancient origin for these mutations, dating to before the divergence of these two species. Some viral-infection-related genes, such as *Il1a* (Pruitt *et al.* 1995; Dinarello 2011) and *Srpk1* (Sciabica *et al.* 2003; Wang *et al.* 2014), exhibited gains of stop codons (Figure 4), which may correlate with the ability of the AHR to serve as a pathogen carrier. The widespread rodenticide, warfarin, can inhibit blood coagulation, and continuous intake of warfarin causes potentially fatal hemorrhages (Davis and Davies 1970; Kohn *et al.* 2000). We found various missense mutations in several genes involved in the warfarin interaction pathway (Figure 4 and File S2), including *Calu*, *Ggcx*, *Ephx1*, *Orm1*, *Pros1*, *Proz*, *Serpinc1*, and *Vkorc1* (I90L). Of note, the I90L mutation in the *Vkorc1* gene can decrease VKOR activity by 10% compared to wild type, which has led to warfarin resistance in rat populations in Argentina (Rost *et al.* 2009; Prescott 2011). These fixed missense SNPs appear to underlie anticoagulant resistance in the AHR (Long *et al.* 2010; Huang *et al.* 2012).

**Figure 5:**
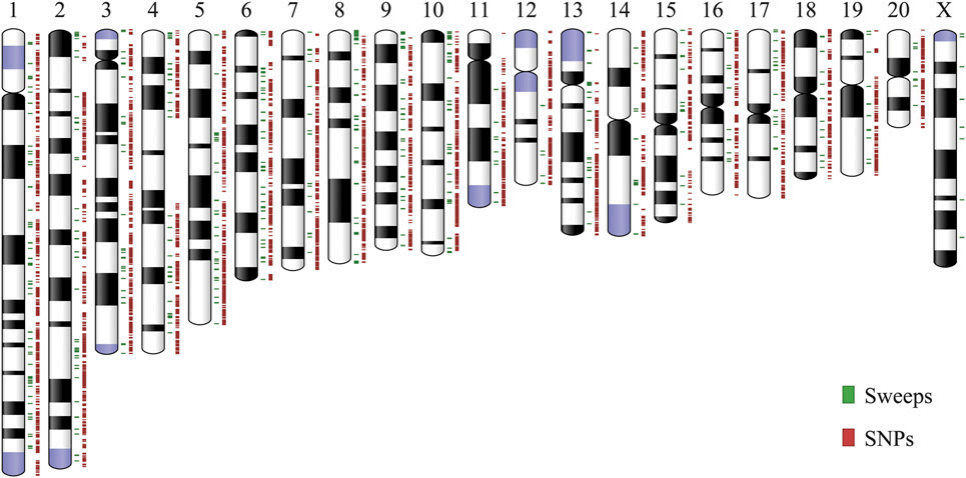
Distribution of selective sweep regions and SNP loci in the AHR. Distribution of AHR selective sweep regions (green) and SNP loci at fourfold degenerate sites (red), according to *R. norvegicus* chromosomes and karyotypes. A sequence gap in the *R. norvegicus* RGSC5.0 reference genome accounts for the region on the chromosome 4 lacking annotation.

Heterozygous neutral SNPs are popular markers for use in assessment of genetic variation in natural populations (Gray *et al.* 2000; Suh and Vijg 2005; Holderegger *et al.* 2006). Through our pooled sequencing experiments, we identified 12,823,195 heterozygous SNPs within the genomes of AHR populations. Due to the degenerate nature of the genetic code, substitutions at fourfold degenerate sites are regarded as neutral variations (Eyre-Walker and Keightley 1999; Nachman and Crowell 2000). We excluded SNPs with high linkage disequilibrium and those residing in duplicated genes from the dataset of heterozygous fourfold degenerate SNPs, resulting in 5800 autosome neutral SNPs in single-copy protein-coding genes (Figure 5 and File S3), providing an ideal panel for future migration or dispersal pattern studies of AHR populations.

### InDels and inversions

**Figure 6:**
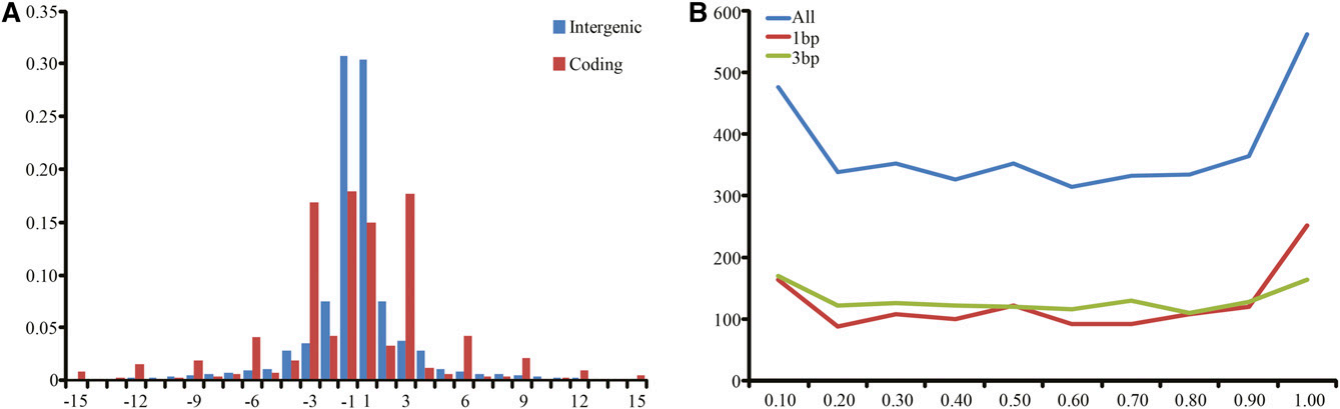
Distribution of InDel sizes in the genome of the AHR. (A) Negative numbers represent deletions and positive numbers represent insertions. (B) The relative locations of InDels within coding sequences are plotted as the first amino acid position of the InDel, divided by the total protein length.

We identified 3,179,903 intraread InDels, with an average density of 1.12 InDels per kb, including 1,624,854 deletions and 1,555,049 insertions. Among all intraread InDels in coding regions, 1244 were 1 bp long, and 1919 were 3n bp long (*i.e.*, the length was a multiple of 3 bp). The proportion of 3n bp intraread InDels in coding regions was significantly higher than that in intergenic regions. In addition, we observed that the InDels within coding sequences were enriched in regions encoding the *N*- and *C*-termini of proteins (Figure 6). Notably, we identified a total of 1881 frameshift InDels, with an allele frequency of less than 0.55 in 1297 genes (File S4). Functional category analyses showed that these genes were related to the cellular response to stress (GO:0033554), sensory organ development (GO:0007423), and regulation of neurotransmitter levels (GO:0001505) (Table S2). Additionally, we found that some genes containing frameshift InDels, such as *Aldh* and *Stard7*, were not single-copy.

**Figure 7:**
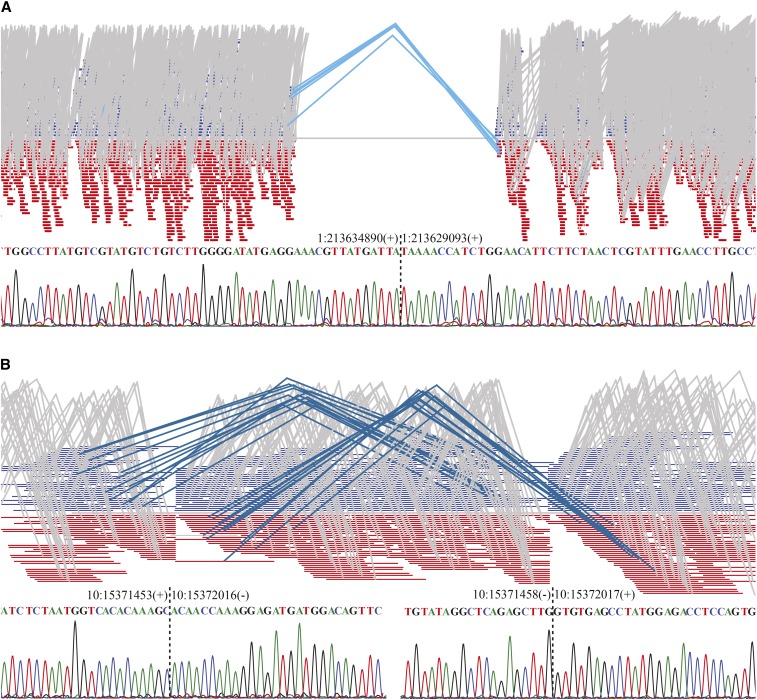
Structural variants in the AHR genome. Representative visualization of read alignments adjacent to (A) deletion and (B) inversion breakpoints in the AHR genome, followed by validation via PCR-based Sanger sequencing. Gray links indicate normally mapped read pairs with proper read orientation and distance. (A) Light blue links represent read pairs with proper read orientation but longer distance, indicating a deletion event in the query sequence. (B) Dark blue links indicate an inversion, causing the paired reads to demonstrate abnormal orientation, with both ends mapping to the same strand.

We detected a total of 12,423 structural variants, including 12,216 deletions, 61 duplications, and 143 inversions (File S5). Using PCR-based Sanger sequencing, we found that the accuracy of the predicted breakpoints was 85% (Figure 7 and File S1). We found an inversion region with a length of 5.46 Mb and different breakpoints (1,357,955:6,822,593; 1,358,196:6,822,593; 1,358,799: 6,821,965; 1,358,402:6,822,135) on chromosome 12. This finding has been confirmed by fluorescence *in situ* hybridization, which showed that the order of the BAC clones on the submetacentric form of chromosome 12 in *R. norvegicus* (12p12, 12p11) was inverted in the acrocentric form of the heteromorphic pairs in the AHR (Badenhorst *et al.* 2011). These chromosomal variations indicate the extensive chromosomal plasticity of the *Rattus* genus.

### De novo assembly of unmapped reads

After assembling the unmapped reads, we obtained 2979 scaffolds longer than 1 kb, with a total sequence length of 3.42 Mb. Computational prediction of gene structure for these scaffolds revealed 20 protein-coding genes. A BLASTP search against the NCBI nonredundant animal protein database revealed that 13 of these genes were homologs of known proteins (E-value <1e-10) (Table S3). Some of these proteins were encoded by motor behavior related genes, such as *Etv1* (Lin *et al.* 1998; de Nooij *et al.* 2013), *SmtnL1* (Wooldridge *et al.* 2008; Lontay *et al.* 2015), and *Npas3* (Erbel-Sieler *et al.* 2004; Brunskill *et al.* 2005), indicating sequence divergence in these genes between the wild AHR and the reference *R. norvegicus* genomes.

### Identification of selective sweeps

Following an integrative analysis of the allele frequency spectrum (Boitard *et al.* 2009, 2012, 2013) and nucleotide diversity of focal regions (Kofler *et al.* 2011) in the AHR population, we detected a total of 570 selective sweeps across 21 chromosomes (Figure 5 and Table S4). Annotation of these regions revealed 1120 genes. Moreover, gene overrepresentation analyses for these regions revealed a relationship with several neural activity related terms, including spontaneous neurotransmitter secretion (GO:0061669), spontaneous synaptic transmission (GO:0098814), positive regulation of synaptic vesicle exocytosis (GO:2000302), and regulation of synaptic vesicle transport (GO:1902803) (Table S5). Transport of synaptic vesicles and the release of neurotransmitters are essential for propagating nerve impulses between neurons. Several genes under positive selection, including *Syt1*, *Unc13b*, *Nlgn1*, and *Stx1b*, are involved in transmission of nerve impulses and synaptic transmission. Glutamate is the major neurotransmitter in the brain, regulating many kinds of behaviors and emotions, and playing an important role in cognitive ability (Petroff 2002). *Gria4* and *Grik1* are important excitatory glutamate receptors, which were also found to be under positive selection in our study. Another family of glutamate receptor genes that were found to be under selection were the inhibitory γ-aminobutyric acid receptors, such as *Gabbr2*. Hence, enrichment of the synaptic function category appears to underlie the evolution of the AHR.

## Discussion

Wild-derived rat species provide novel genome resources that cannot be obtained from the laboratory rat, and that can be used to explore the genetic mechanisms underlying complex behavioral or ecological phenotypes (Smits *et al.* 2005). Here, we report the spectrum of genetic variation of SNPs and SVs across the AHR genome, providing a resource for future genetic studies of the species.

As a highly adaptable pest, AHR has evolved the ability to survive in its ecological habitats. We identified eight fixed missense-mutated genes within the warfarin interaction pathway, which appear to underlie anticoagulant resistance in the AHR (Long *et al.* 2010; Huang *et al.* 2012). In addition to being a notorious pest (Singleton *et al.* 2007; Huang *et al.* 2013), the AHR is a known reservoir of a wide range of human pathogens, including hantaviruses, *Bartonella* spp., and *Leptospira interrogans* (Plyusnina *et al.* 2009; Jonsson *et al.* 2010; Yin *et al.* 2011). Loss of function of several infection-related genes in this species might contribute to its ability to survive in this adverse niche. Given the risk that the AHR constitutes to human health, due to its commensal species and pathogen-carrier characteristics, it is of major interest to investigate the migration and dispersal routes of this species. However, the sensitivity of current molecular markers limits the scope of population research studies. SNPs have been recognized as the markers of choice for population genetics, because of their genome-wide incidence and high frequency, compared to other types of polymorphisms (Collins *et al.* 1997; Landegren *et al.* 1998; Brookes 1999). Heterozygous neutral SNPs are attractive markers for assessment of genetic variation in natural populations (Gray *et al.* 2000; Suh and Vijg 2005), and they have great potential for use in investigation of processes such as gene flow, migration, or dispersal (Holderegger *et al.* 2006). Through our pooled sequencing, we obtained 5800 fourfold degenerate autosomal sites residing in single-copy protein-coding genes (Figure 5). Oligonucleotide hybridization assays or multiplex PCR coupled with NGS sequencing based on these polymorphic neutral sites will enable high-throughput genotyping of multiple individuals. Thus, we have produced an ideal SNP panel for future migration or dispersal pattern studies of AHR populations.

A great number of intraread and large InDels were observed in the AHR genome. We found that the size distribution of intraread InDels (predominantly 1 bp or 3n bp) in coding regions was significantly different from that in noncoding regions, indicating the functional importance of the coding InDels. In addition, we observed that InDels within coding sequences were enriched in regions encoding the *N*- and *C*-termini of proteins (Figure 6). InDels in these regions may be less functionally damaging than those in other coding regions because genes may have alternative translation start sites at the N-terminus or translation of the protein may be almost completed before C-terminal InDels (Ng *et al.* 2008; Pelak *et al.* 2010; Zhan *et al.* 2011). Notably, we found that some genes carrying frameshift variants, such as *Aldh* and *Stard7*, are not single-copy. Due to the functional redundancy of genes in some families, it is conceivable that functional compensation within the same family could rescue the loss of function of a gene containing a nonsense mutation (Teng *et al.* 2013).

Chromosome evolution in rodents has been driven largely by pericentric inversions (Badenhorst *et al.* 2011). An integrative structural-variant caller was used in our study to detect large InDels and inversions (Mohiyuddin *et al.* 2015). Read pairs spanning SV breakpoints produced discordant alignments with an unexpected alignment distance and/or orientation (Chen *et al.* 2009). DNA segments flanking the breakpoint aligned to disjointed locations in split read mapping (Ye *et al.* 2009; Layer *et al.* 2014). This integrative analysis can improve the accuracy of prediction through leveraging multiple orthogonal SV signals, based on both split read and read-pair discordance. The SV calling strategies perform well on mapped regions, although they may miss some SVs in low genome coverage regions. Individuals within populations may have different SV breakpoints in the same region. Due to pooled sequencing, some inversions with different breakpoints were obtained, such as the 5.46 Mb inversions in chromosome 12 (12p12, 12p11). This finding is consistent with previous research (Badenhorst *et al.* 2011). These chromosomal variations indicate the extensive chromosomal plasticity of the *Rattus* genus and provide a rich resource for comparative genomic studies of the rat.

In summary, we report genome-wide SNP and SV variations in the AHR, providing a genomic resource for future studies. Our findings have the potential to contribute to the understanding of the molecular adaptations of the rat to its ecological niches.

## Supplementary Material

Supplemental Material
