# Supplementary material for: Whole-Genome Sequencing Reveals Genetic Variation in the Asian House Rat
Source: G3 (Bethesda). 2016 Apr 27;6(7):1969–77. doi: 10.1534/g3.116.029504 (PMC4938650; doi:10.1534/g3.116.029504)
Supplement: Supplemental Material [file supp_6_7_1969__index.html]

Whole-Genome Sequencing Reveals Genetic Variation in the Asian House Rat — Whole-Genome Sequencing Reveals Genetic Variation in the Asian House Rat — Supplemental Material 

# Whole-Genome Sequencing Reveals Genetic Variation in the Asian House Rat

## Supplemental Material for Teng *et al.*, 2016

**Files in this Data Supplement:**

- File S1 - PCR-based Sanger sequencing of candidate structural variation breakpoints. (.pdf, 627 KB)
- File S2 - Fixed missense mutations in the warfarin interaction pathway of the Asian house rat. (.zip, 3 KB)
- File S3 - Polymorphic and unlinked neutral sites of single-copy protein-coding genes in the Asian house rat genome. (.zip, 290 KB)
- File S4 - Frameshift variants in the genomes of Asian house rat populations. (.zip, 99 KB)
- File S5 - Structural variations in the genome of the Asian house rat. (.zip, 1221 KB)
- Table S1 - Nonsense mutations identified in the genome of the Asian house rat. (.xls, 69 KB)
- Table S2 - Gene ontology analyses of frameshift genes. (.xls, 47 KB)
- Table S3 - Protein predictions from de novo assembled unmapped reads. (.xls, 20 KB)
- Table S4 - Genes in selective sweep regions of the Asian house rat genome. (.xls, 112 KB)
- Table S5 - Functional annotation analyses of selective sweep regions of the Asian house rat genome. (.xls, 303 KB)
