# Supplementary material for: Whole-Genome Sequencing Reveals Genetic Variation in the Asian House Rat
Source: G3 (Bethesda). 2016 Apr 27;6(7):1969–77. doi: 10.1534/g3.116.029504 (PMC4938650; doi:10.1534/g3.116.029504)
Supplement: Supplemental Material [file supp_g3.116.029504_FileS1.pdf]

PCR-based Sanger sequencing of the candidate structural variation breakpoints

The primer sequences of 20 candidate structural variation breakpoints for sanger sequencing

Test ID	Structural variation	Type	Forward primer	Reverse primer
DEL1	11:50549307-50566438	Deletion	TGGTTCTTTCTTCTAAGAGAG	AAACTAATCCTGGGTACATC
DEL2	1:213629093-213634890	Deletion	GTGGCCTCCAAACATATG	CATGCTTTAAGAGGGAGTTC
DEL3	11:92308840-92310743	Deletion	GCAATATACAGCAAACTAACAG	AGACAGAAAGCTGTTTAGGTAG
DEL4	1:262721190-262727474	Deletion	GAGTCGTGATAAACCAAGG	AATTCTTGTGGCTGAACC
DEL5	12:38993403-38993665	Deletion	AACCACTGAGCCATCTCTC	CAAGAGGTCATCAGTGGTG
DEL6	1:38086269-38086923	Deletion	AATACTACTGCTCCTGTTCC	ATAGCACACAGTGAATGTAAAG
DEL7	1:260655415-260661346	Deletion	AATTTGTTTCTGGCACTTC	GAAGAGACTTTGAACTTTGG
DEL8	1:234160312-234174014	Deletion	CTCTGAGCCTCCAGATAAAG	TCAAACCAACCAAAGAGG
DEL9	1:10290542-10305607	Deletion	GTGGTGGCGTTATCAGTAG	GTAAATCCTTCTTCTCCCTG
DEL10	1:13169295-13175227	Deletion	TGATGCCAACTAGCAAGG	TGACCCTTCAGTTACTTCTTC
INV1	5:109309875-109314292	Inversion	GAGAAGAATAGCTAAGAAATCC	GACTTGATGGAGACTCTAAGAG
INV2	10:101908959-101909081	Inversion	CCCTCCTCCTTCTCTTACTC	GGGAGTCTTAGGCCACTTC
INV3	7:54516805-54530515	Inversion	TTCACCCTGTGAGGAGAG	AAACGAATGTAACTGTGTGG
INV4	1:203464083-203464848	Inversion	AGTCAGACCATCCATTCAC	ATATACACCATCGACTCAGTAG
INV5	5:119914323-119916110	Inversion	ATACCATACAACCAGATACAAG	ATTCTATTCAGCTGCAGAG
INV6	11:13008785-13009036	Inversion	AAGAGGCTTACTTTGGTTTC	GCCTAATGAAGTATTTAGCTG
INV7	5:151993298-151993968	Inversion	AGGCATTTGATGAACAGC	GTCCCTGCATGAGAAGTC
INV8	10:15371453-15372019	Inversion	AATAGTTGACCTTACCATGAC	AGATACATGCAGTATTTCAGC
INV9	2:123020464-123022896	Inversion	GCCATTAGACACTGGAGAG	GAGGTGGTATGAGTAATACAAC
INV10	5:115063607-115064257	Inversion	AGTTGGATGACATCAGCC	TTCTGTCTGGCTTGTATACC

Twenty randomly selected deletion and inversion breakpoints were analyzed using polymerase chain reaction (PCR)-based Sanger sequencing with the primers designed according to the de novo assembled scaffolds. For both forward and reverse primer, the sequence was given in 5' to 3' direction.

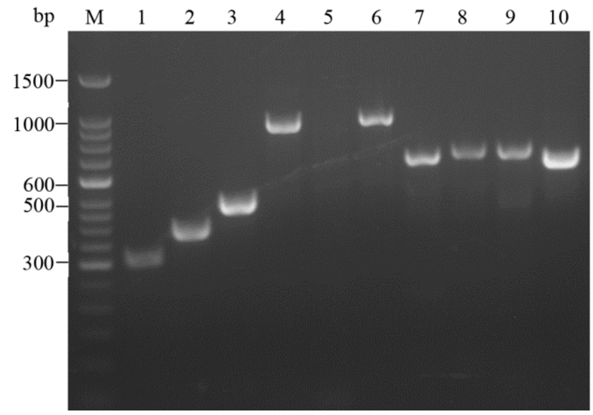

Figure A: PCR results of candidate deletion breakpoints. Lane M: Marker; Lane1–10 represent deletion breakpoints of DEL1–DEL10, respectively.

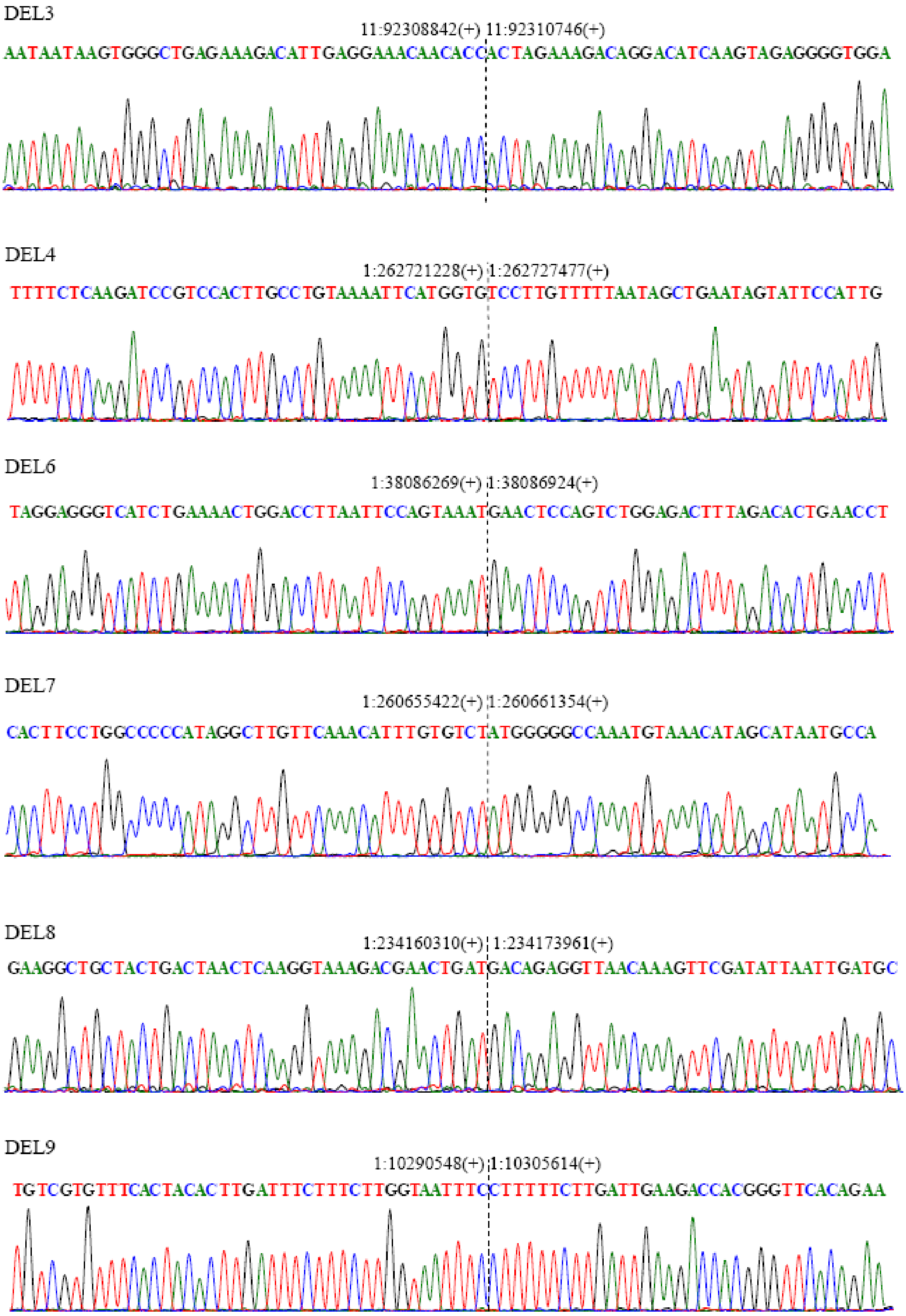

Figure B: Sanger sequencing of candidate deletion breakpoints.

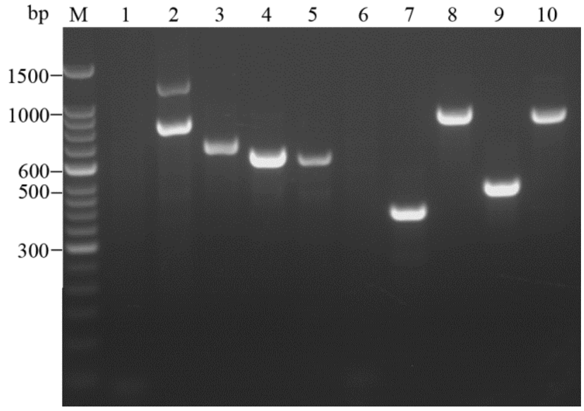

Figure C: PCR results of candidate inversion breakpoints. Lane M: Marker; Lane1–10 represent inversion breakpoints of INV1–INV10, respectively. For large inversions such as INV3, INV4, INV5, INV7, and INV9, only single-end breakpoints were tested.

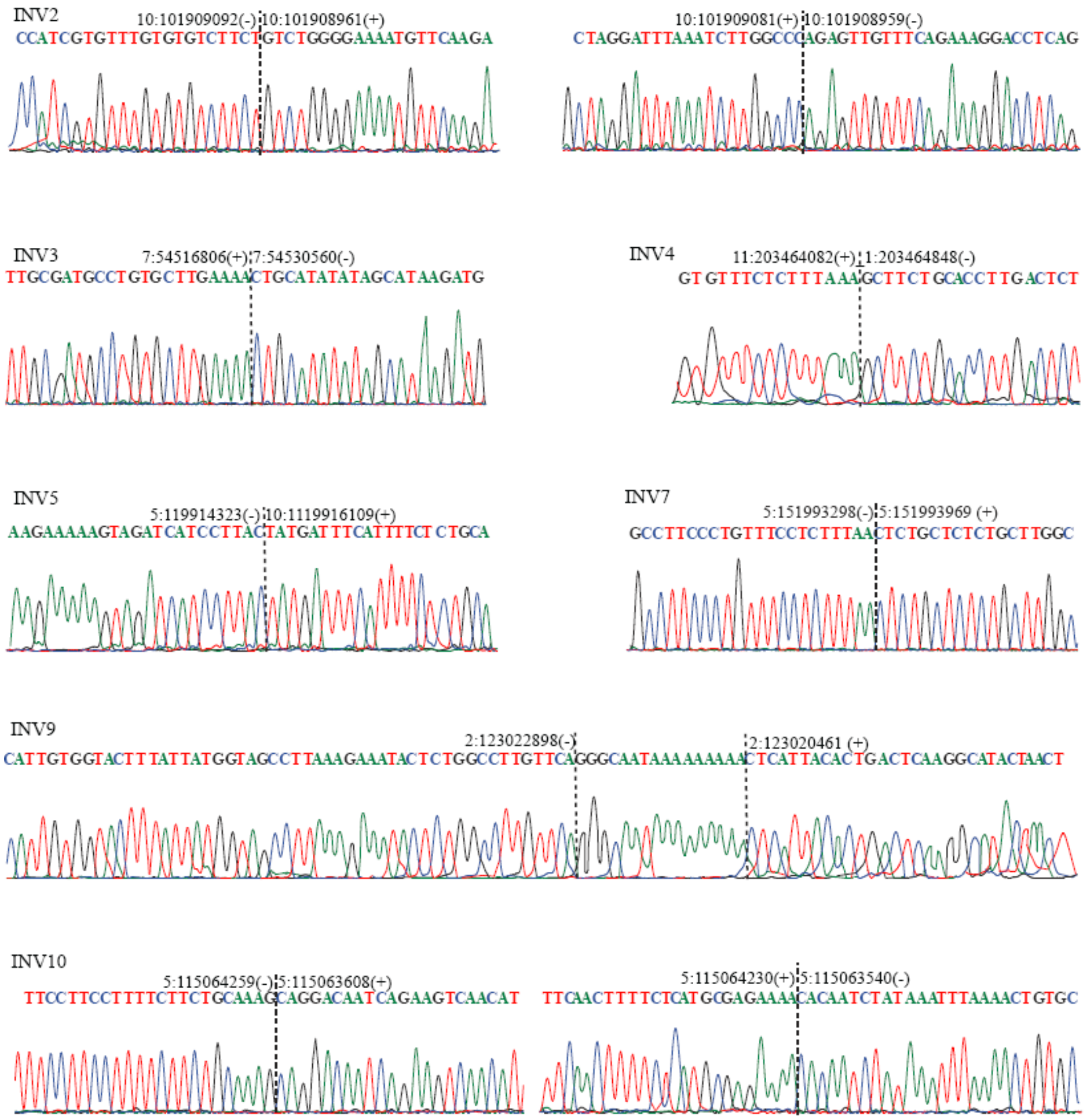

Figure D: Sanger sequencing of candidate inversion breakpoints.
